# Supplementary material for: Screening of leaf extraction and storage conditions for eco‐metabolomics studies
Source: Plant Direct. 2024 Apr 10;8(4):e578. doi: 10.1002/pld3.578 (PMC11004900; doi:10.1002/pld3.578)
Supplement: Supplementary file 1 — Data S1. Peer review [file PLD3-8-e578-s002.pdf]

Plant Direct
Open Access

Screening of leaf extraction and storage conditions for eco-metabolomics studies

Jakob Lang, Sergio E. Ramos, Marharyta Smohunova, Laurent Bigler, Meredith C. Schuman

Decision Letter Round 1:

September 26, 2023

Mr. Jakob Lang
University of Zurich
Zurich, N/A
Switzerland

RE: Screening of leaf extraction and storage conditions for eco-metabolomics studies

Dear Dr. Lang:

Thank you for submitting to Plant Direct. All required reviews have been returned and we have now finished our evaluation of your manuscript. In light of the reviewers' and editor's comments, further revisions are needed before the paper can be accepted for publication in Plant Direct.

Please view the editors' and reviewers' comments below and use their suggestions as a guide while you work on your revision.

When uploading the revised version of this article, please be sure to include the following:

-A word document that contains your response to the reviewers. You should respond to each reviewer comment and note the changes made to the manuscript. If you do not agree with a reviewer's comment and choose not to make a suggested revision, please explain why. Please try to provide as complete an answer as possible to each reviewer's criticisms in the "response to reviewers" section.
-A tracked changes document
- A clean version of the latest version of the manuscript

Comments from Academic Editor:
After careful review, we feel that the submitted manuscript does not meet Plant Direct's publication criteria. We invite you to submit a revised manuscript that carefully addresses each of the points outlined by the reviewers and academic editor, and a revised version may be considered for publication.

These points include:

1. Expand the Introduction to included cited background on tissue storage and metabolite extraction procedures that are commonly employed in the field or lab setting.

2. Reorganize the manuscript and Figure 1 to improve clarity, logic and flow. Clearly describe and differentiate between the different combinations of extraction method, storage temperature and storage duration. Acronyms should be defined and method names should be used consistently across sections, figure legends, etc. The terms, broad method and narrow method screening need to be clearly defined or renamed. Results for each combination should be clearly presented and appropriate figures and supplemental figures cited. See Reviewer 2 comments.

3. Provide a thorough analysis of the metabolite data to assess whether specific metabolite classes are differentially extracted using the different methods. Present statistical analyses (e.g. for individual metabolites and metabolite classes) and comparisons amongst the extraction methods. Metabolite data should also be included as a supplementary table. See comments from Reviewers 1 and 2 for specific suggestions.

4. Properly cite and summarize relevant literature and protocols in the introduction and discussion.

To upload your revision, please click the link below.
https://plantdirect.msubmit.net/cgi-bin/main.plex?el=A5Lr6bz6A5oKt5I5A9ftd3lSR8L8y1XSHDfnHBhm9QZ

In order to provide as timely a service as possible, we ask that your revision is resubmitted within three months after receipt of this request. If an extension is needed, please send a request, along with a brief explanation, to the editorial office at plantdirect@wiley.com .

Thank you very much for giving us an opportunity to review your work. I look forward to receiving the next version.

Sincerely,
Marna Yandeau-Nelson

-- Editor comments:
After careful review, we feel that the submitted manuscript does not meet Plant Direct's publication criteria. We invite you to submit a revised manuscript that carefully addresses each of the points outlined by the reviewers and academic editor, and a revised version may be considered for publication.

These points include:

Plant Direct
Open Access

1. Expand the Introduction to included cited background on tissue storage and metabolite extraction procedures that are commonly employed in the field or lab setting.

2. Reorganize the manuscript and Figure 1 to improve clarity, logic and flow. Clearly describe and differentiate between the different combinations of extraction method, storage temperature and storage duration. Acronyms should be defined and method names should be used consistently across sections, figure legends, etc. The terms, broad method and narrow method screening need to be clearly defined or renamed. Results for each combination should be clearly presented and appropriate figures and supplemental figures cited. See Reviewer 2 comments.

3. Provide a thorough analysis of the metabolite data to assess whether specific metabolite classes are differentially extracted using the different methods. Present statistical analyses (e.g. for individual metabolites and metabolite classes) and comparisons amongst the extraction methods. See comments from Reviewers 1 and 2 for specific suggestions.

4. Properly cite and summarize relevant literature and protocols in the introduction and discussion.

-- Reviewer comments:
Reviewer #1:

This is a great manuscript by Lang et al. which provides some answers to questions many researchers in ecometabolomics have wondered but none (to my knowledge) has really answered. It definitely deserves publication and will help ecologists make decisions on how to handle their valuable samples from the field to the lab. I still have a few comments which may help improve the manuscript:
Lines 51-55 : I would give more details about the drying processes available to researchers. Lyophilisation is the gold standard since it theoretically prevents enzymes to work during the drying process and best maintains phytochemical integrity. However, lyophilisers are usually not available at field sites either. The researcher is thus left with either oven-dried, air-dried or silica gel-dried options which all induce strong drought stress to the plants and may greatly modify the metabolomes. Hence the need for alternatives such as solvent-based solutions used in the present study.
Line: 158: refer to figure S4.
Line 180: does CSA ionize well in positive ionization as well or is it restricted to negative ionization?
Lines 205-206: I would mention that this is perfectly logical since DCM extracts lipids and other hydrophobic molecules while SPE eluted with MeOH will mostly keep them.
Line 230: although a trend to the right is already visible for 4{degree sign}C samples.
Figure 5 is very interesting but, if possible, I would go deeper into metabolite annotation to further interpret Fig 5B. Which metabolite classes account for the differences observed? For instance I can imagine that benzoxazinones may be lost during air drying but potentially conserved by LLE and liquid N2 treatments. In addition, air-drying might convert glycosylated flavonoids to aglycones or at least remove a sugar unit from flavonoids with multiple sugars. Using the TIMS-TOF PASEF data it should be possible to annotate a number of compounds and get additional information on the

specific metabolome changes that have occurred during the different processes.
Also, in Figure 5D, and as mentioned lines 232-234, we can clearly see an effect of the storage time at -20{degree sign}C after shock-freezing in liquid N2 (at least until 28 days, then samples seem to stabilize). This suggests that storing extracted samples in solution is better in terms of stability than keeping fresh frozen tissues at -20{degree sign}C. Yet, it would be interesting to do the same experiment for samples stored at -80{degree sign}C. I know this is not really the point here since the paper is intended to ecological studies where it is rarely possible to keep samples below -80{degree sign}C uninterruptedly, but I would be very curious to see if a similar trend is observed or if frozen samples are more stable at a lower temperature. Maybe the authors could add a sentence about it after line 234 or in the discussion.
Figure 6: I would remove the 28 days samples of the FE-SPE approach and remove the sentence lines 256-258 too. Also check if the sentence lines 254-256 still applies after removing 28 days points.

Reviewer #2:

1. The data presented in Fig.5B appears to be the main piece arguing that the on-site extract prepared samples behave similarly to the flash frozen samples. However, this data has not been fully explored. While it is true PC1 shows the airdry samples are different from the extract and LN2 samples, PC2 shows that LN2 and airdry directly overlap and the extract samples are mostly different. Another concern about PC2 is the large within-group variation for the LN2 samples, which I believe is supposed to be the laboratory standard procedure. It appears that the variation within the LN2 group is greater than the between-group variation. Also, PC2 still accounts for 16.3% of the variation, making it an important factor to account for. Some explanation must be provided for what is going on with this PC. Finally, PC3-5 are mentioned, but no data is provided on them at all. A scree plot showing the five PCs would be nice to see in the supplemental.

2. It was hard to keep track of all the various extraction methods, timepoints, and temperatures. Therefore, consistency between text and labels of figures is crucial. For instance, is the LLE optimisation also the narrow method screening? Line 146 of SI, is the Narrow Screening Method the same as the LLE optimisation in Fig.1B?

3. There is a lack of references throughout the text. Considering that this manuscript focuses on expanding methodology, the older techniques utilized should be well referenced to support your use of them. Specifically, the following three areas should be addressed at a minimum if possible:
a. Should include references to support the basis of the main extraction method chosen.
b. References to support the SPE extractions would also be good to include.
c. References or other reasoning to back up the initial choice of the three internal standards selected should be included to help strengthen your use of them.

4. Considering that this paper is about differences in extraction methods, it would be a good idea to include somewhere in the text details about why the different extraction methods were selected or how they compare fundamentally.

5. Many of the supplemental figures are not even referenced in the text. They should at the very least be linked to something in the text or otherwise removed from the manuscript.
a. For instance, I believe that figures S8, S9, S10, S11, S12, and S13 could be referenced in the sentence spanning lines 222-225
b. The sentence starting in line 225 should be rewritten to focus only on Figures S6 and S7.
c. Fig. S14, S15, S17, and S18 need to be referenced in the text as well at appropriate spots.
d. Fig.S16 and the methyl jasmonate treatment should be briefly mentioned in the results section.

6. Lines 226-234 should be reworked. Based upon the sentence starting in line 226, the reader is led to believe that fig.5A is about the metabolite profile shifting over time when it is about storage temperature. fig.5C and 5D show changes over time.

7. Lines 268-278, what data corresponds to these claims? There are no referenced figures.

8. Is the Paragraph at line 156-165 referring to work completed in the lab, if so, this should be explicitly stated somewhere for clarification.

9. Line 12 of SI, handing should be handling.

10. SI 1.2.3, I do not understand why this section is called "Leaf Storage (Leaf)". All of the sample preparation methods include some form of leaf storage. Doesn't this section include the laboratory standard procedure? That should be highlighted here.

11. SI section 1.2 should be presented in the order displayed in Fig. 1A. The figure and text would then work together in helping the reader keep track of all the extraction methods.

12. Lines 167-169 of SI, was there some extraction solution added here?

13. Fig.S4, There is an A, B, C, D, but these are not discussed in the figure caption.

While PCA is a great visualization tool for looking at the data, it does not provide much insight into what is actually changing in the results. A suggestion would be to explore the possibility of including additional analyses. Are some metabolites or classes of metabolites showing up or disappearing with certain methods? Are recovered abundances changing? Do certain extractions work better for some classes of molecules over others? A heatmap could provide another nice visualization of the data for specific metabolites or broad classes. Conducting some statistical tests on the actual data, not just PC components, would also help strengthen the findings. This wouldn't have to be done on all the data presented in the study. Fig.5B could serve as reason to further explore the comparisons observed there.

Plant Direct
Open Access

Decision Letter Round 2:

February 23, 2024
Mr. Jakob Lang
University of Zurich
Zurich, N/A
Switzerland

MSID: 2023-01250-TWR2
MS TITLE: Screening of leaf extraction and storage conditions for eco-metabolomics studies

Dear Dr. Jakob Lang:

I am pleased to inform you that your manuscript "Screening of leaf extraction and storage conditions for eco-metabolomics studies" has been accepted for publication in Plant Direct.

Your article will appear online in the next available issue of Plant Direct. To ensure your article gets published as quickly as possible, please pay attention to the steps detailed below. We have found that most of the delays happen at this stage, especially at the payment stage, so please respond as quickly as possible when prompted.

License Agreement: Once your article has been accepted it will move to Production and undergo admin and file checking - you may receive an email with any queries we have at this stage. When all required items are received by the publisher and queries resolved, the corresponding author will receive an email from Wiley's Author Services system which will ask them to log on at https://authorservices.wiley.com/bauthor and will present them with the appropriate license for completion. Your article cannot be published until both the signed license agreement and payment of the article fee have been received.

Payment of the Open Access Article Publication Fee: All articles published in Plant Direct are fully open access: immediately and freely available to read, download and share. Plant Direct charges a publication fee to cover the publication costs. The corresponding author for this manuscript should have already received a quote from the payments team (cs-openaccess@wiley.com) with the estimated article publication fee; please email cs-openaccess@wiley.com if this has not been received. The corresponding author should log on to the Wiley Author Services site, where the publication fee can be paid by credit card or an invoice. Pro Forma can also be requested. Payment of the publication charge must be received before the article will be published online.

Proofs: You will have the opportunity to look over your paper once more when you receive the author proofs for your article. The proofs will be with you in approximately two weeks. Please note that, in addition to publishing reviewer comments, the author's responses to review comments will also be published alongside the final version of the paper. If you would not like the author's responses to be published, please contact the editorial office at plantdirect@wiley.com .

Promotion of your article: You can help your research get the attention it deserves! Check out Wiley's free Promotion Guide for best-practice recommendations for promoting your work at www.wileyauthors.com/eeo/guide. And learn more about Wiley Editing Services which offers professional video, design, and writing services to create shareable video abstracts, infographics, conference posters, lay summaries, and research news stories for your research at www.wileyauthors.com/eeo/promotion.

Thank you again for your contribution to Plant Direct. If you have any questions, feel free to contact the editorial office at plantdirect@wiley.com .

Sincerely,

Marna Yandeau-Nelson

Hsou-min Li

Editor, Plant Direct

-- Editor comments
Thank you for addressing the reviewer's comments by clarifying normalization procedures and adding additional normalization results into the manuscript. We are pleased to accept this manuscript for publication at Plant Direct.

-- Reviewer comments:

Author Responses:

Replies to the reviewers

Editor comments:

After careful review, we feel that part of the manuscript needs further clarification. We invite you to submit a revised manuscript that clarifies the normalization process that was used and specifically addresses the concern raised by Reviewer 1 regarding the need to normalize the data separately from the positive and negative modes.

Reply to the Editor:

We appreciate the opportunity to further clarify the normalisation process to further improve the manuscript. We modified parts of the manuscript (lines 201-206 and 281-287) and parts of the

supplementary information (section 3.3) to better describe the normalisation process and the main limitation of our approach. We added Fig. S22 to visualise the impact of choosing a different normalisation approach. We further took the chance to edit axis labels and titles of the figures 4, 5, 6, and 7 in the manuscript to improve readability. Lastly, some minor edits were done across the manuscript to more be more consistent with the use of abbreviations.

Reviewer comments:

Reviewer #1:

Overall the authors have done a great job at answering the reviewers' questions. I particularly appreciate the fact that they expanded on metabolite classes annotation as requested by both reviewers. However, there is still one major point which in my opinion was not correctly addressed. It is related to my initial question "1.4 Line 180: does CSA ionize well in positive ionization as well or is it restricted to negative ionization?"

The authors' reply is: "Occasionally CSA can be found in positive ionization mode, primarily as an [M+H-H2O]+ ion. This is a low-abundance and high-variance ion, however which would cause more harm than good when used for normalization. As an internal standard for positive ionization mode, our lab has used ampicillin in the past, but we have concerns about its long-term stability, and recently we tried stevioside, which has the mentioned in-source fragmentation issues. Since both of those compounds have notable downsides, we decided to proceed with normalization based on CSA's signal in negative ionization mode."

I actually wanted to know if CSA could be used to normalize positive ion data, which is obviously not the case. I must apologize for not having been more explicit in my original comment. Unless I missed something, the authors acquired data in positive and negative modes separately, merged the datasets and normalized all the data to the intensity of CSA obtained in negative mode (except for one dataset which was only acquired in positive mode and where the NH4+ adduct of stevioside was used). This would work only if positive and negative ionization data were simultaneously acquired. Here the variation induced by injection volume, signal decrease (or increase) over time etc. cannot be accounted for in the positive ion dataset, and it is thus not an acceptable way to normalize the data. Normalization is a complex process which is still under debate within the metabolomics community and has not found an absolute consensus. I leave it to the authors to get more insights on the topic and possibly find more suitable internal standards for future studies (although I am not a big fan of normalization to internal standards myself). Yet, in the present case, I can think of three normalization options which would be far better than normalization to a single internal standard in a single ionization mode:

1. Integral normalization (i.e. to the total spectrum)
2. Normalization to a selected set of identified metabolites present in all samples. This option often outperforms integral normalization since it eliminates the small noisy peaks which pollute the spectra.
3. Probabilistic quotient normalization (PQN)

The normalization step must be separately performed on both positive and negative datasets before merging. This will certainly not modify the results, but at least represent a robust way to normalize data which cannot be criticized by the readers.

Reply to Reviewer #1:

We appreciate the valuable input on the normalisation methods for our dataset. We are aware that this is a debated topic with no clear consensus yet, and normalisation was one of the main points of discussion within our team before starting the experimental work.

Firstly, we realised that our manuscript was evidently not clear enough when describing the normalisation used for the data that assesses the stability across the storage duration. The normalisation used signals of stevioside for both storage experiments: the broad screening (only positive mode) used the ammonium adduct, the LLE Optimisation data (positive and negative mode) was normalised using the ammonium $[M+NH_4]^+$ and formate $[M+COOH]^-$ adduct signals of stevioside. We adjusted the manuscript Section "Suitability of Internal Standards", lines 201-206 in the new version, and SI Section 3.3 to clarify that the dataset was normalised on a signal of stevioside.

Our goal for the normalisation by internal standard is primarily to adjust for variation originating from sample handling and transport. This is in our experience the major contributor to intra-sample variation, especially when performing extractions without proper laboratory infrastructure. The sample handling and transport errors can be assumed to affect the positive and negative data equally, which means a normalisation by internal standard in one mode can be used for the other mode as well to account for sample handling. This was further clarified in the section "Suitability of internal standards" (lines 201 – 206)

Our main concern with using the total spectrum normalisation is that in our experience it works best to compensate for dilution effects and instrument-based fluctuations of samples which are very similar to each other with differences limited to a small subset of metabolites. Once the samples are more diverse, the normalisation can artificially introduce differences in metabolite levels between sample groups that are not representative of the real concentration in the samples. A large difference in the overall sample profile is something we for example observed during a study of farm-grown maize plants, where plants occasionally were beginning senescence, showed severe drought stress, or severe herbivory. Those plants unsurprisingly had a very diverse overall metabolite profile where differences were already obvious from a visual comparison of the base peak chromatogram. Even though these plants are highly diverse, some metabolites still show a comparable signal across all samples, but a normalisation across the total spectrum would introduce differences in those signals. We do not have that much experience with probabilistic quotient normalisation (PQN), but it seems to be susceptible to similar issues as total spectrum normalisation.

We want the method shown in this manuscript to be capable of handling samples with diverse metabolite profiles. To handle this challenge, we consider normalisation on a known stable signal to be the most logical approach and using an internal standard for that purpose is the simple option. The normalisation on a set of identified and unchanging metabolites you mentioned would be the more challenging version as it requires previous knowledge of stable and unchanging metabolites. To

work for our experimental conditions, we would have needed to identify compounds that remain at a constant level even during early senescence, droughts and after herbivore attacks. Finding such a set of metabolites and proving that they maintain a constant level under those conditions is a challenging task, which is why we chose the simpler internal standard normalisation.

Nevertheless, following the reviewer's comment, we tested the normalisation on the total integral and PQN. We found that the outcome does not fundamentally change but did see some minor changes in the sample distribution. Therefore, we edited the manuscript section "Extract stability over time" (lines 281 – 287) to reflect those minor shifts. Additionally, we now include Fig.S22 which shows the difference in the PCA distribution following probabilistic quotient normalisation. All of this shows that for the dataset presented in this study, PQN or total spectrum normalisation is an adequate method to handle the data. Due to the points discussed above, we still want to highlight normalisation based on internal standard as our recommendation to remove data fluctuations originating from sample handling and transport.

Reviewer #2:

The additions to the manuscript helped improve the clarity and flow, which was much appreciated. It is clear which extraction methods, storage temperatures, and storage durations are being discussed throughout the text. All figures are now cited in the text which greatly improves data interpretation. Relevant literature and protocols are cited in both the introduction and discussion.

The compound classifications were a great addition to the manuscript, helping to strengthen the findings. However, there are still no new statistical analyses on the metabolite data. The MANOVA on the PCAs is okay for telling us that there are differences in the extractions, but does not directly address the question of whether specific metabolites are changing. These types of analyses are quite common in metabolomics data, even datasets larger than this. There are ways to pare down the size of large datasets as well, and tools such as MetaboAnalyst will even do this for you. It is disappointing to not see statistical analyses like these utilized, but the manuscript has greatly benefited from the addition of compound classes at the very least.

Line 323, compare should be compared.

Reply to Reviewer #2:

Thank you for the positive evaluation of the changes we implemented. We consider that additional statistical analyses are more appropriate when the focus of the study is on a specific class of metabolites. As our study was designed to show the influence of extraction methods on a broad range of metabolites, we think that our statistical methods are sufficient to provide an overview of metabolite profile changes resulting from different methods. Readers who have a specific molecule in mind will be able to statistically test whether their metabolites of interest change depending on extraction and storage methods in the dataset we provide on Zenodo.

Line 323 was changed, thank you for the comment.
